# Navigating the AI revolution: challenges and opportunities for integrating emerging technologies into knowledge management systems. Systematic literature review

**DOI:** 10.3389/frai.2025.1595930

**Published:** 2025-07-04

**Authors:** Teona Gelashvili-Luik, Peeter Vihma, Ingrid Pappel

**Affiliations:** ^1^Department of Software Science, Tallinn University of Technology, Tallinn, Estonia; ^2^Ragnar Nurkse Department of Innovation and Governance, Tallinn University of Technology, Tallinn, Estonia

**Keywords:** artificial intelligence, emerging technologies, knowledge management, organizational performance, digital transformation

## Abstract

**Introduction:**

Artificial intelligence (AI) is transforming organizational knowledge management (KM) by leveraging techniques such as machine learning, neural networks, and fuzzy logic to enhance knowledge discovery, capture, storage, and sharing. While this shift promises improved efficiency and personalization, it also poses challenges related to data quality, employee resistance, and alignment with existing workflows.

**Methods:**

This study presents a systematic literature review (SLR) of 40 peer-reviewed publications focused on the integration of AI in KM. The review follows PRISMA guidelines and includes thematic coding to identify patterns, critical success factors, and knowledge gaps.

**Results:**

Findings indicate that successful AI-enabled KM depends on strong leadership commitment, adaptable governance structures, and context-sensitive technology selection. AI’s role is evolving from supporting routine tasks to enabling dynamic, real-time knowledge flows. The review also highlights a critical need to balance automation with human oversight.

**Discussion:**

Key gaps were identified in understanding cost–benefit trade-offs, ethical implications, and governance mechanisms. These insights suggest directions for future research focused on practical, accountable, and empirically validated KM strategies. As part of an ongoing research project, the synthesized findings will inform the design of future empirical studies. The evidence suggests that, when strategically implemented, AI can serve as a competitive enabler in knowledge-driven organizations.

## 1 Introduction

Knowledge Management (KM) has long been recognized as critical to organizational performance and long-term competitiveness (Harrington et al., 2019; Manesh et al., 2020; Foli, 2022). Contemporary KM extends beyond intra-firm activities to encompass regional and multi-stakeholder ecosystems, fostering innovation across diverse sectors (Weck et al., 2022; Muzzio and Gama, 2024). Recent research has expanded its focus to include organizations of various sizes, external knowledge flows, and broader geographic contexts (Shekhar and Valeri, 2023; Castagna et al., 2020).

Knowledge Management Systems (KMS) form the technological and organizational infrastructure for capturing, storing, sharing, and applying knowledge. In the digital era, KMS convert dispersed data into actionable insights, enabling collaboration and innovation (Alavi and Leidner, 2001; Davenport and Prusak, 1998). The integration of artificial intelligence (AI), big data analytics, and cloud computing has further advanced these capabilities, helping organizations manage complexity and pursue strategic objectives (Jarrahi et al., 2023; Georgiev and Antonova, 2024). Nevertheless, many firms still struggle to realize the full potential of these technologies, highlighting a persistent research and practice gap (Herrero et al., 2016; Kaplan and Haenlein, 2019).

The rise of Industry 4.0—the digital transformation of industrial processes—has further increased interest in the intersection of KM and emerging technologies (Li et al., 2019; Babkin et al., 2019). To remain competitive, organizations increasingly focus on knowledge lifecycle management, digital infrastructure, and human-centered strategies (Gupta et al., 2022).

AI is playing a growing role in optimizing business processes and generating data-driven insights (Beheshti et al., 2021; Esposito et al., 2024). Applications such as data mining, predictive analytics, and supply chain optimization illustrate this trend (Mahmood, 2019; Khan and Vorley, 2017; Hashem et al., 2024; Torres-Dela Cruz et al., 2019). However, the broader strategic and organizational implications of AI integration into KM remain underexplored.

While several systematic reviews have examined IT and AI impacts on KM (Al Mansoori et al., 2021; Ofosu-Ampong, 2024; Samuels, 2025), gaps remain regarding the specific technologies involved, their organizational consequences, and the implementation barriers encountered. This review seeks to address these gaps by synthesizing current literature on the integration of AI and emerging technologies into KMS, assessing their impact on KM practices, and identifying challenges and strategies for effective implementation.

Accordingly, this study is guided by the following research questions:

- RQ1: How do AI and emerging technologies impact organizational KM practices?
- RQ2: What are the primary challenges in updating existing KM processes to match current trends?
- RQ3: How can AI and emerging technologies be leveraged to address these challenges?

To answer these questions, a Systematic Literature Review (SLR) methodology was employed, following established guidelines to ensure methodological rigor and minimize bias (Kitchenham, 2004). The SLR enables a structured synthesis of key trends, insights, and gaps across diverse scholarly sources.

The article is structured as follows: Section 2 reviews KM development and identifies key research gaps; Section 3 outlines the methodology; Section 4 presents the findings and discussion; and Section 5 concludes with limitations and directions for future research.

## 2 State of the art

This chapter reviews key developments in knowledge management research. It traces the field’s evolution from foundational concepts through the impacts of digitalization and AI, ending with a summary of literature gaps that motivate this study.

### 2.1 Early discussions and principles of KM implementation

Organizational performance has long been a central focus in management research, emphasizing leadership (Bass and Riggio, 2005), organizational culture (Cameron and Quinn, 2011), and innovation adoption (Damanpour, 1998; Rogers, 2003; Pacheco and Paul, 2023). Knowledge management emerged as a critical factor for enhancing performance by managing information, knowledge, and experience to extend organizational capabilities (Nerney, 1997; Skyrme and Amidon, 1997; Mayo, 1998; Bassi, 1997).

Early KM research identified key organizational and technical challenges in implementation (Lloyd, 1996; Davenport, 1997), emphasizing the importance of integrating human networks with technology. Davenport et al. (1998) highlighted several pillars of successful KM: operational foundations such as infrastructure and flexible knowledge structures; cultural facilitators including a knowledge-friendly environment and motivational practices; optimized knowledge flow through multiple transfer channels supported by leadership; and economic integration. Bennett and Gabriel (1999) further linked formal KM procedures to increased innovation, adaptability, and improved access to knowledge.

From the 2000s onward, technological advances inspired research into digitalization’s impact on enterprise knowledge networks and lifecycle management (Vladova et al., 2018; Babkin et al., 2019). Open innovation perspectives expanded KM systems to incorporate emerging technologies like the Internet of Things (Santoro et al., 2018) and frameworks for organizational knowledge visualization were also proposed (Smuts and Scholtz, 2020). This progression reflects a shift from foundational KM concepts toward integrating innovation, digitalization, and organizational dynamics.

### 2.2 Digitalization and human-centric approaches in modern KM research

During this time, knowledge management has developed in two main ways: advances in technology and a focus on people. Digital tools have changed organizational operations by enabling quicker decisions and supporting new ideas (Radavičius and Tvaronavičienė, 2022; Vladova et al., 2018). Using digital systems for knowledge management helps make workflows more efficient and can improve overall organizational results (Schäffer et al., 2021).

At the same time, human-centered approaches focus on the role of people, social interactions, and organizational culture in creating, sharing, and using knowledge. Supporting ongoing learning that meets different employee needs is key to effective knowledge management (Viterouli et al., 2023; Castellani et al., 2021; McIver and Lepisto, 2017). Encouraging active knowledge sharing based on teamwork and human judgment plays an important part in fostering innovation (Muzzio and Gama, 2024). Social and relational aspects of knowledge transfer—which cannot be fully captured by digital tools—remain essential to success (Nguyen et al., 2023; Retkowsky et al., 2024). Sharing tacit knowledge through face-to-face interaction and communities of practice continues to support learning and innovation within organizations (Kamasak et al., 2017; Obembe and Obembe, 2020).

Effective knowledge management combines technology with attention to people’s experience and insights. Digital tools help organize and share information efficiently, while human involvement is essential to capture the knowledge that cannot be easily documented. Balancing these aspects is important as organizations adopt new technologies without losing expertise held by their employees (Malik et al., 2021).

### 2.3 Advancements in AI-driven knowledge management

Recent advances in artificial intelligence have led organizations to adopt more sophisticated technologies in knowledge management. Data mining methods—such as neural networks and decision trees—are now used to reveal hidden knowledge, improve forecasting, and support decision-making (Bandaru et al., 2017; Tsai, 2013; Natek and Zwilling, 2014). AI also enhances knowledge transfer and sharing, and contributes to building expert systems through machine learning and semantic technologies (Jia et al., 2012; Abubakar et al., 2019; Alonso et al., 2012; López-Cuadrado et al., 2012; Herrero et al., 2016).

Research has expanded from focusing solely on organizational performance to also considering wider societal issues. Organizations increasingly acknowledge how digital knowledge management tools affect employee well-being, job performance, and access to knowledge (Babkin et al., 2019; Ferraris et al., 2017; Castellani et al., 2021). Recent studies examine human factors like trust, attitudes toward information technology, interpersonal behaviors, and leadership’s influence on knowledge sharing (Castellani et al., 2021). Additionally, research investigates how technology adoption impacts mental health and well-being, with organizational support, such as training and leadership, playing a moderating role (Nguyen et al., 2023). Sustainability has also become an important topic, with knowledge management framed as a strategy to secure and sustain competitive advantage (Gupta et al., 2022).

### 2.4 Emerging trends in knowledge management research

The evolution of knowledge management is reflected in academic research, with literature reviews adapting to new developments. Studies such as Inkinen (2016) and Radavičius and Tvaronavičienė (2022) have focused on KM digitalization, while others examine knowledge creation, transfer, and digital innovation (Smuts and Scholtz, 2020; Di Vaio et al., 2021). Research has also highlighted links between KM, digital transformation, and Industry 4.0 (De Bem Machado et al., 2022). Recent reviews have broadened their scope beyond technology to include human-centered topics, such as cognitive support (Li et al., 2019) and adult learning theories within organizational culture (Viterouli et al., 2023).

Despite the extensive discussion on KM digitalization, integrating AI into traditional KM is still underexplored. Some scholars propose AI-focused KM frameworks that combine human and technological elements (Fteimi and Hopf, 2021), while others investigate KM challenges in remote and hybrid work environments (Taherdoost and Madanchian, 2023). However, research remains limited, and recent systematic literature reviews highlight the need for further research (Al Mansoori et al., 2021; Ofosu-Ampong, 2024).

Many existing reviews examine these topics from a narrow angle, often relying on a single database. For example, Inkinen (2016) and Radavičius and Tvaronavičienė (2022) used Scopus, Li et al. (2019) used Web of Science, and Shekhar and Valeri (2023) and Al Mansoori et al. (2021) relied on ScienceDirect. While these databases are reputable, focusing on only one may miss relevant studies found elsewhere.

This review aims to provide a thorough analysis of themes, concepts, and findings across the KM field by searching multiple databases and applying no restrictions on time, publication type, or source. This approach seeks to reduce the risk of overlooking important research.

### 2.5 Theoretical foundations of knowledge management

The empirical and technological advances discussed above are grounded in established theoretical frameworks that explain how knowledge is created, shared, and utilized within organizations. Understanding these foundational models is essential for interpreting the evolution of KM practices and the impact of emerging technologies.

The evolution of knowledge management has been shaped by several influential theories. The SECI model (Nonaka and Takeuchi, 1995) conceptualizes knowledge creation as a dynamic process involving socialization, externalization, combination, and internalization, emphasizing the interplay between tacit and explicit knowledge. The Dynamic Capabilities Framework (Teece, 2007) highlights an organization’s ability to sense, seize, and reconfigure resources in response to change, positioning knowledge as a key dynamic asset. Furthermore, Distributed Cognition (Hutchins, 1995) and Organizational Learning (Argyris and Schön, 1978) focus from individual or centralized knowledge to systems where knowledge is constructed and enacted through ongoing interaction among people and technologies. These frameworks provide a lens for analyzing how successive technological paradigms in KM (from expert systems to generative AI) reflect changing assumptions about how knowledge is created, shared, and leveraged in organizations.

## 3 Methodological applications

This systematic literature review (SLR) follows established guidelines from Kitchenham (2004) and Webster and Watson (2002), ensuring transparency, rigor, and reproducibility. The review process included: (1) formulation of research questions, (2) development of a comprehensive search strategy, (3) application of predefined inclusion and exclusion criteria, (4) data extraction, and (5) thematic synthesis. The Preferred Reporting Items for Systematic Reviews and Meta-Analyses (PRISMA) framework (Liberati et al., 2009) guided the reporting of the study selection process.

### 3.1 Search strategy and resources

Search terms were developed in direct alignment with the research questions and study objectives. To ensure comprehensive coverage, the search strategy incorporated a broad set of keywords and their commonly used synonyms, including variations in terminology used across disciplines. This approach was designed to reduce the risk of omitting relevant studies that may use different descriptors for knowledge management, artificial intelligence, or related emerging technologies. The search string was refined through an iterative process involving preliminary testing and adjustment, ensuring both sensitivity and specificity in capturing pertinent literature. Boolean operators (AND, OR, NOT) were applied to structure the search logic and to connect key concepts effectively. The final search string used was: (“knowledge management technology” OR “knowledge management tools” OR “knowledge management processes”) AND (“intelligent systems” OR “emerging technologies” OR “digitalization” OR “artificial intelligence”) AND “organization” AND (“adoption” OR “drivers” OR “strategies” OR “challenges” OR “success factors”) AND “innovation” NOT (“public sector” OR “government”).

To maximize coverage and minimize bias, we searched four major academic databases: Scopus, Web of Science, ScienceDirect, and Google Scholar. No restrictions were applied regarding publication date, type, or language. Reference lists of selected papers were manually screened to capture additional relevant studies. Where direct export was not possible (e.g., Google Scholar), bibliographic details were manually entered into a master spreadsheet.

### 3.2 Study selection

The PICOS framework, which is widely recognized for structuring eligibility criteria in systematic reviews (Higgins and Green, 2011; Schardt et al., 2007), informed inclusion and exclusion criteria:

- Population: Organizations using or implementing KM systems
- Intervention: AI, digitalization, or emerging technologies applied to KM
- Comparison: Traditional KM or alternate technological approaches (where applicable)
- Outcomes: Impact on KM processes, organizational challenges, and strategic responses
- Study Design: Empirical studies, SLRs, and theoretical papers published in peer-reviewed journals or conferences

Study selection proceeded in two phases: (1) screening of titles and abstracts, and (2) full-text assessment. Inclusion and exclusion criteria are summarized in Table 1.

**Table 1:** Inclusion and exclusion criteria.

Inclusion criteria	Exclusion criteria
Relevance: Studies focusing on the relationship between KM and organizational performance.	Relevance: Studies not directly relevant to the intersection of KM, AI, and emerging technologies in organizational settings.
Impact on knowledge processes: Studies addressing the impact of digitalization, AI, and emerging technologies on KM practices.	Focus: Studies focusing solely on the public sector or government organizations.
Challenges and strategies: Studies examining the challenges and strategies for updating existing KM processes to align with current trends.	Specificity: Studies do not address the specific research questions outlined in the Introduction section.
Variability across industries/sizes: Studies exploring variations across industries and organizational sizes.	Non-English literature: Non-English studies.
Language: Studies published in English language.	

### 3.3 Data analysis and quality assessment

**Figure 1:**
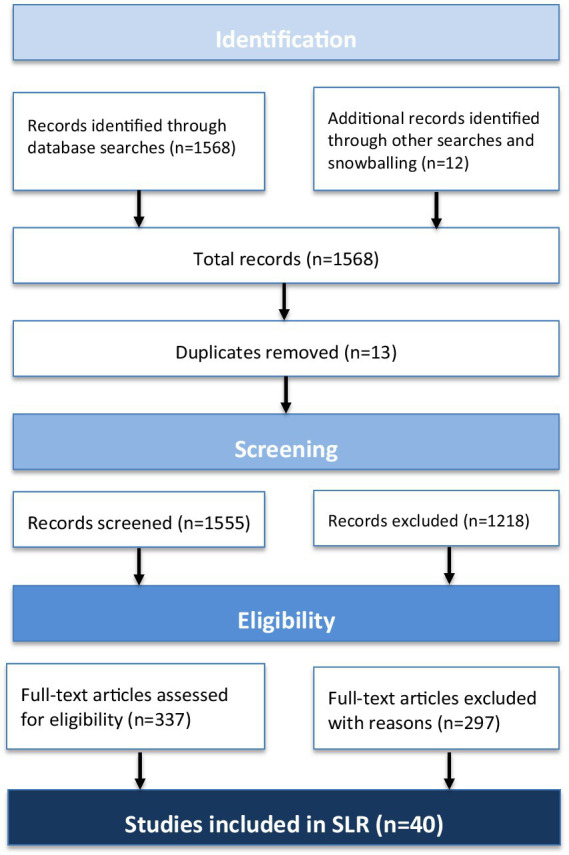
PRISMA flow diagram illustrating the study selection process.

A total of 1,568 records were identified through searches in Scopus (266), Web of Science (21), Google Scholar (1100), ScienceDirect (169), and additional manual searches (12). After removing duplicates, identified based on matching titles, authors, and publication years, 1,555 records remained. The screening process involved two stages: initial title and abstract screening, which reduced the pool to 337 records, followed by a full-text review based on predefined eligibility criteria. Ultimately, 40 studies are included in the review. The detailed selection process is illustrated in the PRISMA flow diagram (Figure 1).

The selected articles were subjected to thematic analysis following the approach outlined by Nowell et al. (2017). During the coding phase, key concepts and findings relevant to the research questions were identified and organized into categories. These categories were then synthesized into overarching themes reflecting the impact of AI and emerging technologies on knowledge management practices, associated challenges, and strategic responses. The inclusion of studies from diverse geographic regions, publication years, and disciplinary perspectives helped mitigate potential biases related to narrow focus or source reliance.

Study quality was assessed using a customized matrix based on the CASP checklists (Critical Appraisal Skills Programme, 2018) and adapted for mixed-methods studies. The matrix included seven criteria as presented on the Table 2, each scored on a 0–2 scale (2 = fully met; 1 = partially met; 0 = not met). Studies were classified as high (scores of 12–14), medium (8–11), or low quality (0–7), using thresholds informed by CASP-based scoring approaches and the Mixed Methods Appraisal Tool (MMAT) framework (Hong et al., 2019).

**Table 2:** Quality assessment criteria.

Criterion	Description
1. Clarity of aims	Are the research objectives clearly stated and aligned with the study design?
2. Methodology appropriateness	Is the methodology (qual/quant/mixed) justified and suitable for the aims?
3. Data collection transparency	Are data collection methods described in sufficient detail?
4. Analysis rigor	Is the analysis process transparent, systematic, and reproducible?
5. Bias consideration	Does the study address potential biases or limitations?
6. Relevance to SLR focus	How directly does the study address AI/emerging tech in KM?
7. Technological context	Does the study adequately describe the AI/emerging technology used (e.g., NLP, machine learning)?

Quality assessment was conducted in accordance with the predefined criteria outlined in the quality assessment matrix. Based on these criteria, 80% of the studies were rated as high quality, 15% as medium quality, and 5% as low quality (for quality scores for each study see Supplementary Appendix B). The final selection of studies included in the review was reached by consensus among all authors.

Studies rated as relatively low quality (overall score of 7) were early-stage conceptual papers. While these studies are vital for understanding the development of the field, the primary analysis and outcomes presented in the following chapters are based on the high-quality publications.

### 3.4 Methodological limitations

This review only includes peer-reviewed, published literature in English, which may introduce publication and language bias. Although multiple databases were searched, some relevant studies may have been missed due to terminology variation or manual data entry errors.

The search string was designed to capture a broad spectrum of relevant studies. However, its complexity may have inadvertently excluded articles using alternative or less common terms. Balancing comprehensiveness and precision in search strategies remains an inherent challenge in systematic reviews.

Screening, coding, and thematic analysis involve elements of subjective judgment. While clear criteria and established methods were applied to minimize bias, some degree of interpretive subjectivity is unavoidable. The heterogeneity in study designs, contexts, and methodologies among the included articles may also influence the comparability and generalizability of the findings.

## 4 Results and discussion

This chapter addresses research questions, with each sub-section focused on a specific question. Table 3 provides a summary of the main technology categories and their contributions to knowledge management, based on the 40 studies included in this review (for detailed study-level information see Supplementary Appendix A). Following the summary, a more in-depth analysis of the publications is presented, exploring each AI or emerging technology and its associated research area within knowledge management.

**Table 3:** Summary of technology categories and their contributions to knowledge management.

Technology/category	Representative studies (Year)	Study type(s)	Main KM process(es)	Key insights
Generative AI and AI	Retkowsky et al. (2024), Stollberg et al. (2004), Iaia et al. (2024), Stohr et al. (2024), Safadi and Watson (2023), Leoni et al. (2022), Schäffer et al. (2021), and Jia et al. (2012)	Empirical, conceptual, technical	Knowledge transfer, sharing, creation, retention, collaboration, structuring	AI and generative models (e.g., ChatGPT) transform knowledge work, collaboration, and sharing, frameworks and empirical studies highlight risks, mechanisms, and implementation guidance.
Neural networks and hybrid AI	Liebowitz (2001), Kawonga et al. (2023), Sanders et al. (2019), Herrero et al. (2016), and Miradi et al. (2009)	Empirical, conceptual, technical	Knowledge capture, discovery, diagnosis, integration, competitiveness	Neural networks and hybrid AI support knowledge discovery, scaling, and decision support across industries.
Fuzzy logic and machine learning (ML)	Yadegari and Mohammadi (2024), Grzeszczyk (2021), Anshari et al. (2023), and Vladova et al. (2018)	Empirical, conceptual, technical	KM system modeling, collecting, sharing, discovery	Fuzzy logic and ML enhance KM system modeling, knowledge collection, and automation
Big data and data mining	Shaqrah and Alzighaibi (2023), Abualoush (2025), Sumbal et al. (2021), Thomas and Chopra (2020), Safhi et al. (2019), Khan and Vorley (2017), Depeige and Doyencourt (2015), Gullo (2015), Mahmood (2019), Natek and Zwilling (2014), Nguyen et al. (2014), and Alonso et al. (2012)	Empirical, conceptual, technical	Acquisition, sharing, discovery, application, workflow optimization	Big data and data mining drive knowledge acquisition, discovery, and application, especially in healthcare, and education.
Other digital technologies	Wielgórka (2023), Kane (2017), Bandaru et al. (2017), Ristoski and Paulheim (2016), Bianchi et al. (2016), Braun et al. (2016), Santoro et al. (2018), Pisoni et al. (2024), and (Leoni et al., 2024)	Empirical, conceptual	KM capacity, innovation, collaboration, knowledge integration	Internet of Thing (IoT), cloud, social media, and analytics tools foster innovation, collaboration, and digital knowledge integration in diverse sectors.

### 4.1 AI and emerging technologies impact on organizational KM practices

This sub-section answers the first research question by analyzing how artificial intelligence and emerging technologies are changing core knowledge management functions. To explore this transformation of how knowledge is created, distributed, and applied within organizations, the section is divided into thematic sub-categories, each focused on a distinct technological domain.

#### 4.1.1 Data science and analytics

The rapid growth of organizational data presents both strategic opportunities and operational challenges. Big Data Analytics (BDA) provides powerful tools to process this data, but without mechanisms to convert it into actionable knowledge, its strategic value remains limited (Rialti et al., 2020). Knowledge management serves as a critical bridge between data and decision-making, ensuring that insights translate into informed organizational decisions. Artificial intelligence and machine learning are central to this process. Automated data mining and real-time extraction tools support the generation of knowledge in action. For example, Finogeev et al. (2017) demonstrate how AI, integrated with distributed computing infrastructures such as cloud and fog systems, processes sensor data in real time to support operational decisions. Likewise, Ristoski and Paulheim (2016) show how Semantic Web technologies connect heterogeneous datasets to create integrated, context-aware knowledge systems.

Data science reshapes each phase of the KM lifecycle. In creation, machine learning uncovers patterns that would be difficult for humans to detect (Nguyen et al., 2014). For storage and retrieval, semantic technologies and classification algorithms enhance organization and accessibility. Knowledge sharing is becoming personalized through analytics that tailor content to user needs, while application is supported by dashboards and predictive models embedded directly into workflows. More than enhancing each KM phase, BDA reconfigures the entire process. Rather than a linear sequence—create, store, share, apply—KM becomes a dynamic feedback loop. Knowledge is treated as provisional, continuously updated based on new data. This recursive model allows past applications to inform future knowledge through real-time monitoring and learning mechanisms.

One forward-looking approach is Knowledge-Driven Optimization (KDO), which uses knowledge generated during processes to improve future performance (Bandaru et al., 2017). This supports adaptive, self-correcting systems. It reflects a shift from knowledge-as-asset—a static, codified resource—to knowledge-as-flow, where value lies in relevance and responsiveness. In this flow-based KM model, knowledge is continuously generated, revised, and embedded in real-time interactions among systems, algorithms, and decision environments.

This shift challenges traditional frameworks such as Nonaka and Takeuchi (1995) SECI model, which emphasizes human-centric knowledge creation, particularly through socialization and tacit knowledge exchange. In contrast, flow-based KM repositions the human actor as peripheral, privileging algorithmic pattern recognition and system-level feedback. While SECI views knowledge as emerging through reflection and conversion, flow-based KM treats it as emergent, iterative, and embedded in automated systems.

This transformation also introduces significant governance challenges. When knowledge is continuously evolving, how can organizations ensure its trustworthiness, accuracy, and accountability? Algorithmic decision-making often obscures the origin and rationale behind knowledge outputs, raising both technical and epistemological concerns. Consequently, KM must now incorporate real-time processes for curating, validating, and explaining knowledge.

AI, data science, and analytics extend beyond operational functions to challenge traditional assumptions about knowledge itself. Is knowledge objective and stable, or inherently dynamic and distributed? While these questions warrant further exploration, these technologies clearly elevate knowledge as a strategic organizational asset. They facilitate real-time insights, tailor knowledge flows to individual needs, and embed intelligence directly into processes, while simultaneously demanding new approaches to knowledge governance and understanding.

#### 4.1.2 Computational intelligence

Computational Intelligence (CI), which includes neural networks, fuzzy logic, and evolutionary algorithms, supports knowledge management by allowing systems to learn from data, adjust to new information, and function in uncertain conditions. In contrast to traditional AI approaches that rely on predefined rules, CI is better equipped to address the complexity and ambiguity often present in organizational knowledge systems.

A key contribution of Computational Intelligence in knowledge management is its alignment with the Dynamic Capabilities Framework. This framework outlines three core capabilities necessary for organizations operating in uncertain environments: sensing opportunities and threats, learning from experience, and responding effectively (Teece, 2007). CI techniques support sensing by analyzing diverse and incomplete data to identify relevant patterns; they enable learning through the iterative refinement of knowledge models; and they support response by integrating decision-making tools into organizational processes. In this way, CI helps shift KM from a primarily static repository function toward a more adaptive, real-time process of organizational learning and informed action.

Herrero et al. (2016) illustrate this shift with their Hybrid Artificial Intelligence System (HAIS), which identifies KM weaknesses and generates adaptive insights, supporting flexible and ongoing KM assessment. Similarly, Delen et al. (2013) emphasize how CI enhances knowledge utilization by learning from user behavior to recommend contextually relevant content, ensuring timely and effective application within daily decision-making.

CI supports the integration of fragmented and tacit knowledge across organizational functions. For example, Grzeszczyk (2021) presents a fuzzy logic framework for processing unstructured documents, illustrating how CI can enable automated knowledge extraction and targeted dissemination. Such approaches support more adaptive and flexible knowledge management systems, moving beyond static repositories and enabling real-time responsiveness to evolving informational needs.

Collectively, these developments suggest that CI extends the Dynamic Capabilities Framework by making knowledge itself a continuously evolving capability—one that is sensed, learned, and enacted in real time through intelligent systems embedded within organizational processes. However, the adoption of CI in KM presents challenges. Issues such as algorithmic interpretability, bias in training data, data quality, and lack of transparency can undermine trust and accountability. These risks highlight the need for governance structures to ensure ethical, explainable, and responsible CI use in KM, balancing technological potential with critical oversight.

#### 4.1.3 From expert systems to AI assistants

The development of artificial intelligence in knowledge management reflects a shift from static, rule-based systems toward more flexible and adaptive approaches. Early KM technologies operated on fixed logic and emphasized codified knowledge, whereas current AI-enabled systems support more personalized access, the use of tacit knowledge, and decisions that respond to contextual nuances. This section introduces a four-stage framework that outlines how successive waves of AI technologies have shaped and redefined KM practices over time.

##### 4.1.3.1 Stage 1. Rule-based expert systems (1980s–1990s)

Expert systems relied on explicitly coded “if–then” rules to simulate expert-level decision-making within defined domains (Liebowitz, 2001). These systems supported basic automation and were effective in environments where knowledge could be clearly articulated and structured. However, they lacked adaptability and were unable to deal with uncertainty, change, or the nuanced, tacit knowledge that characterizes many organizational processes. As a result, their applicability remained narrow and domain-specific.

##### 4.1.3.2 Stage 2. Ontology-driven and NLP-enabled KM (2000s)

The early 2000s introduced systems that integrated ontologies and natural language processing to enable more sophisticated, user-friendly knowledge retrieval. Platforms such as h-TechSight (Stollberg et al., 2004) reflected a move toward semantic KM, improving flexibility in categorizing and accessing knowledge assets. While these systems enhanced the organization and discoverability of knowledge, they still relied on largely static architectures with limited capacity for real-time learning or adaptation.

##### 4.1.3.3 Stage 3. Predictive and adaptive KM systems (2010s)

Advancements in machine learning, big data analytics, and user modeling enabled KM platforms to become more predictive and adaptive. These systems could analyze behavior patterns, infer user intent, and deliver personalized knowledge recommendations. As Delen et al. (2013) noted, even sophisticated infrastructures fail if knowledge is not usable. Predictive systems helped overcome this by contextualizing content and enhancing knowledge applicability. The adaptive nature of these systems made them increasingly valuable in dynamic environments, where relevance and timeliness are essential.

##### 4.1.3.4 Stage 4. Interactive and generative KM systems (2020s)

The current generation of KM technologies is characterized by real-time, interactive platforms operated by conversational and generative AI. Tools such as ChatGPT and IBM Watson engage users in natural language dialog, facilitate the extraction of tacit insights, and support collaborative knowledge creation (Retkowsky et al., 2024). These systems embed knowledge directly into workflows, reduce users’ cognitive load, and promote sensemaking across organizational contexts. Their capacity to learn from interaction and adapt to context reflects a major shift toward human–AI co-production of knowledge.

As outlined in the State of the Art (Section 2.5), these technological shifts reflect evolving theoretical perspectives in KM. Table 4 presents a summary of each stage’s technological paradigm, knowledge focus, and organizational role, building on these foundational frameworks. This theoretical progression highlights how the dominant knowledge focus and organizational impact have evolved alongside underlying KM theories.

**Table 4:** Evolution of AI in knowledge management: technological and theoretical shifts.

Period	Technological paradigm	Knowledge focus	Organizational impact	Theoretical orientation
1980s–1990s	Expert systems (Rule-based KM)	Explicit, codified knowledge	Task automation, decision support	Codified knowledge, stability, SECI
2000s	Ontology and NLP (Semantic KM)	Metadata, structured texts	Structured access, retrieval improvement	SECI, early dynamic capabilities
2010s	Machine learning and predictive analytics (Adaptive KM)	Tacit and contextual knowledge	Adaptive reasoning, real-time feedback	Dynamic capabilities, organizational learning
2020s	Conversational AI and generative models (Embedded KM)	Cognitive processes, interaction, tacit and explicit knowledge	Human–AI collaboration, embedded knowledge assistance	Dynamic capabilities, organizational learning, distributed cognition

### 4.2 Primary challenges in updating existing KM processes

This next part answers second research question by examining the challenges preventing organizations from effectively updating their KM processes in response to emerging AI technologies. Instead of treating these challenges as separate issues, the analysis highlights how technical constraints, organizational resistance, and poor strategic alignment often interact and reinforce one another.

#### 4.2.1 Data quality and integration

The increase in organizational data volume and diversity places considerable pressure on KM systems and exposes critical vulnerabilities in data quality, consistency, and integration. While heterogeneous data sources ranging from structured databases to unstructured social media content offer rich knowledge potential, they simultaneously complicate seamless knowledge flow (Kawonga et al., 2023; Ristoski and Paulheim, 2016). This fragmentation complicates the process of turning raw data into practical knowledge, increasing the risk of inefficiency and poor strategic coordination. Therefore, the growth in data volume might become a liability instead of an asset. As data volumes increase, it often becomes more difficult to maintain clarity and practical usability, which can hinder effective interpretation and decision-making. This highlights the importance of determining whether there is a practical limit to the amount of data that can meaningfully contribute to insight before it leads to information overload or misalignment.

Legacy IT systems often reinforce data silos, where knowledge remains locked within departments or platforms, making it difficult to access or reuse across the organization (Kawonga et al., 2023). This fragmentation limits the ability of AI-driven KM tools to deliver value, as these tools depend on integrated, high-quality data to function effectively. Efforts to improve interoperability, such as using Semantic Web technologies like Linked Open Data (LOD), offer potential solutions, but their practical application faces significant barriers. In many cases, organizations rely too heavily on a few central knowledge bases, limiting coverage and relevance. Additionally, inconsistent standards and uneven adoption across systems reduce the benefits of these technologies (Ristoski and Paulheim, 2016). This reflects on a critical challenge in knowledge management when new tools are often introduced without fully addressing the constraints of the existing IT environment. It can leading to partial solutions that fail to scale and result in an ongoing tension between technological ambition and infrastructural capacity.

The high speed and diverse formats of big data present challenges for maintaining quality throughout the knowledge discovery process (Safhi et al., 2019). In practice, organizations often struggle to validate, clean, and standardize incoming data quickly enough for it to be useful. Choosing the right analytics tools is not just a technical matter, it directly affects how well knowledge can be extracted and applied. Poorly chosen frameworks or weak sensor data management can lead to processing errors, misinterpretations, and unreliable outputs (Kawonga et al., 2023; Finogeev et al., 2017). If these issues are not resolved, they affect the entire knowledge management system by reducing the reliability and relevance of the insights produced. In this context, data quality and integration are not just technical concerns but directly influence the trustworthiness and timeliness of decisions.

#### 4.2.2 Organizational and human factors

Technological upgrades to KM systems often encounter friction coming from human and organizational dynamics. Resistance toward AI-based tools is often caused by fears of job displacement and skepticism toward automation which reveals deeper cultural and psychological barriers to change (Herrero et al., 2016; Lei, 2022). This resistance slows adoption and hinders organizations from successfully applying KM to support learning, knowledge exchange, and innovation.

A misalignment between an organization’s knowledge management maturity and the complexity of new technologies can further complicate the adoption process (Herrero et al., 2016). Without a clear assessment of organizational readiness and a tailored change management approach, implementations are more likely to underperform or fail. Gaps in digital literacy and limited training opportunities may hinder user engagement and reduce the effective use of new technologies, adding to these challenges (Miradi et al., 2009; Obembe and Obembe, 2020).

Tacit knowledge capture which is embedded in individual experience and to codify it remains one of the most persistent challenges in knowledge management. AI-enabled tools, such as natural language processing systems or conversational agent, offer potential solutions, but their success depends heavily on user trust, participation, and alignment with organizational culture (Obembe and Obembe, 2020). One-size-fits-all KM strategies often overlook sector-specific and cultural variations, leading to uneven adoption and ineffective knowledge sharing (Delen et al., 2013).

For organizations aiming to improve their readiness for knowledge management initiatives, it is important to evaluate cultural absorption capacity alongside technological infrastructure and financial resources. Human factors, such as attitudes toward change, openness to collaboration, and engagement with new systems, should not be viewed merely as barriers. Instead, they represent key enablers that can significantly influence the long-term effectiveness and adaptability of KM efforts.

#### 4.2.3 Organizational size and resource constraints

Organizational size, its structural capacity and resource availability influences the implementation and outcomes of knowledge management initiatives. Larger organizations, including multinational corporations (MNCs), often operate with complex hierarchies, departmental segmentation, and legacy information systems that inhibit efficient knowledge flow (Harrington et al., 2019). In such contexts, KM interventions typically require formal governance structures and comprehensive technical frameworks to ensure integration across divisions and geographies. In contrast, small and medium-sized enterprises (SMEs) may benefit from flatter organizational structures and more flexible decision-making processes, which can facilitate the adoption of KM practices (Kianto et al., 2018; Harrington et al., 2019). However, SMEs frequently face limitations in digital infrastructure, technical expertise, and financial capacity, which restrict their ability to implement and sustain advanced AI-based KM systems as well as their long-term maintenance and user-training (Wielgórka, 2023).

Given these differences, KM strategies must be adapted to organizational scale and resource profiles. In larger firms, the emphasis lies in system interoperability, data governance, and cross-functional alignment. In smaller firms, effective KM requires low-cost, user-friendly tools that do not exceed existing operational capacity. A uniform approach to AI integration across organizations of varying size is therefore unlikely to produce equitable outcomes.

#### 4.2.4 Governance and ethical concerns

As AI and big data become more central to knowledge management systems, questions of governance and ethics become core concerns. One of the main challenges is the lack of transparency in how AI systems make decisions. When algorithms produce results that users cannot explain or understand, it becomes difficult to trust the system (Safadi and Watson, 2023). Without clear rules for how data is used, and decisions are made, people’s resistance in adopting increases which in the end limits tools’ usefulness in knowledge management.

In addition to trust, legal and ethical risks must be considered. Organizations are responsible for protecting sensitive data, complying with regulations, and ensuring that data is not misused (Schäffer et al., 2021; Leoni et al., 2022). A failure to do so can result in more than legal fines, it can lead to reputational damage, employee resistance, or even financial losses. Strong governance structures must therefore go beyond compliance and promote a shared understanding of ethical data use across the organization.

What complicates these challenges further is the rapid pace of technological innovation. Emerging tools frequently introduce capabilities that existing governance models were not designed to address. In many cases, ethical shortcomings arise not from deliberate misconduct but from outdated policies or ambiguous accountability structures. This emphasizes a critical issue: whether static governance frameworks remain adequate in contexts where technologies evolve continuously. There is a growing need to consider whether governance mechanisms themselves must become more dynamic and responsive, capable of evolving in parallel with the tools and risks they aim to regulate.

If governance is to become adaptive, it raises questions regarding the assignment of responsibility. Determining who is accountable for updating governance frameworks and ensuring the ethical and transparent use of new technologies is essential. In the absence of clearly defined roles and processes, oversight risks becoming fragmented, inconsistent, or altogether overlooked. Thus, governance should not be viewed merely as a compliance function, but as a continuous, distributed responsibility that shapes how knowledge management systems are implemented, trusted, and sustained within organizational contexts.

#### 4.2.5 Technological complexity, scalability and integrating KM approaches

Integrating AI and big data tools into knowledge management is rarely straightforward. Many existing KM systems were built around traditional methods focused on storing and retrieving explicit knowledge, rather than handling the volume and speed of new data sources or supporting AI-driven analysis (Rialti et al., 2020; Sumbal et al., 2021). Updating these systems involves complex technical choices about architecture, data infrastructure, and ongoing maintenance that directly affect how reliable and usable the KM system is day-to-day (Kawonga et al., 2023).

A common issue is that organizations often treat traditional KM processes and emerging AI-driven methods as separate, which limits their ability to combine the strengths of both (Rialti et al., 2020; Sumbal et al., 2021). Traditional KM usually follows linear steps of capturing, storing, and sharing explicit knowledge while big data analytics works through iterative, exploratory processes aimed at discovering patterns and creating knowledge in real time (Sumbal et al., 2021). This difference means organizations need integrative frameworks that balance the steady, controlled flow of traditional KM with the flexible, dynamic nature of AI-based knowledge discovery.

The ability to implement and scale these complex solutions varies widely. Large organizations typically have the technical expertise and resources to adapt and expand AI-enhanced KM systems. Smaller or less digitally mature organizations often lack these resources, leading to a “scalability gap” where some firms move forward while others lag behind, potentially increasing inequality in knowledge capabilities for (Shaqrah and Alzighaibi, 2023; Abualoush, 2025).

This gap makes it essential the KM tools to be designed for different organizational contexts. For example, machine learning algorithms can speed up data mining and knowledge creation (Nguyen et al., 2014), but their effectiveness depends on how well they are adapted to the specific context highlighting that automation cannot fully replace human judgment or domain expertise.

Addressing technological complexity and scalability goes beyond technical fixes. It requires organizations to rethink how they integrate traditional KM and AI-driven approaches, and to plan for different levels of capacity and maturity. Failing to do so risks fragmented knowledge flows, underused data assets, and missed out innovation opportunities.

#### 4.2.6 Technological complexity, scalability and integrating KM approaches

**Figure 2:**
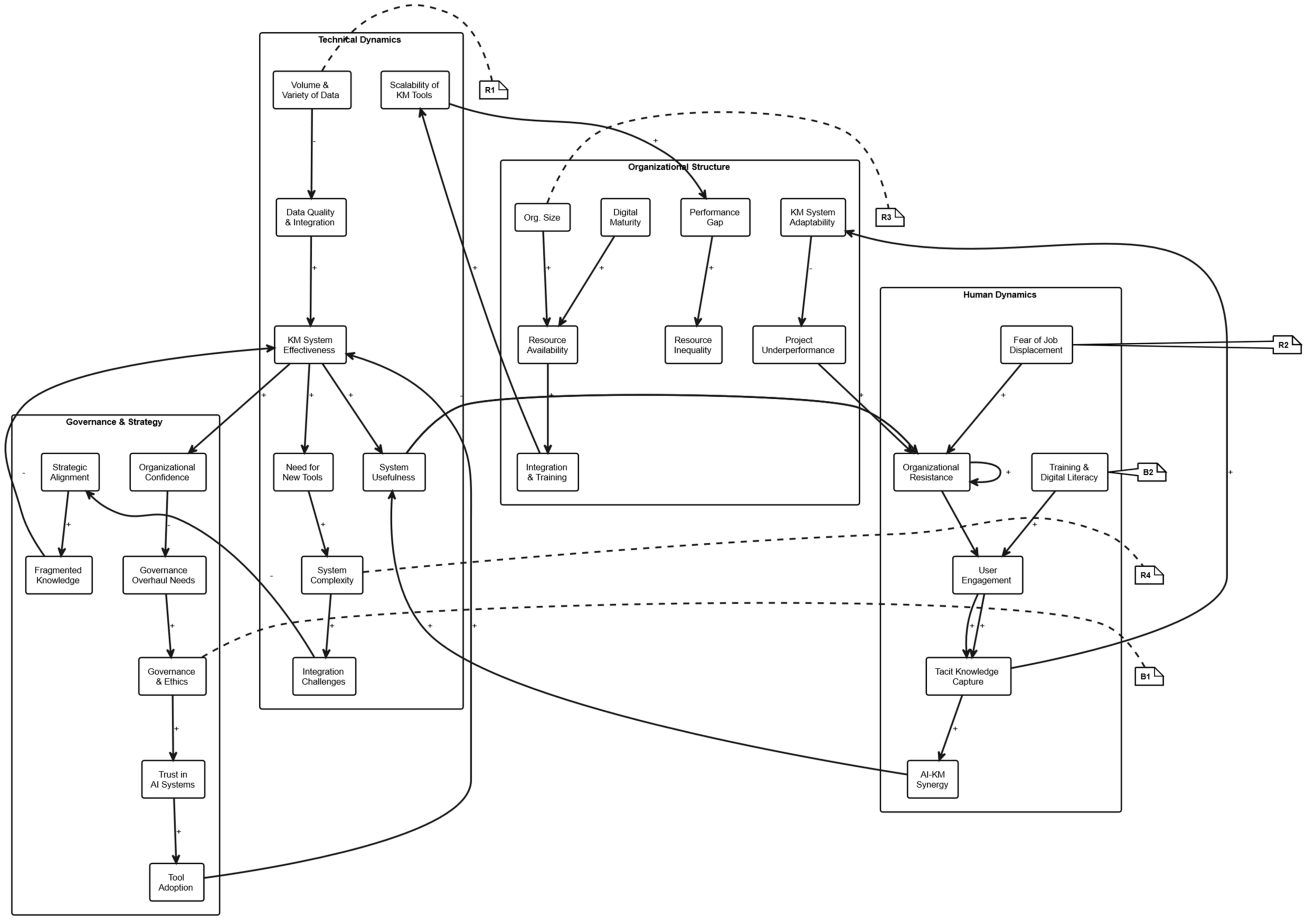
Causal loop diagram.

To better understand the interdependencies highlighted in the previous sections shaping AI-enabled knowledge management systems, this study incorporates a Causal Loop Diagram (CLD) (see Figure 2). The CLD visualizes the dynamic feedback relationships among key technological, human, organizational, and governance variables that influence the performance, scalability, and sustainability of KM initiatives. It maps elements such as data quality, legacy IT systems, resistance to change, AI adoption, trust, digital literacy, and governance capacity, showing how these interact through reinforcing and balancing feedback loops.

At its core, the model reveals several critical systemic dynamics:

- The Data Chaos Loop (R1) illustrates how rapidly increasing data volume and heterogeneity—when inadequately integrated—can erode data quality. This in turn weakens AI tool performance and KM effectiveness, leading to misinterpretation of outputs, organizational distrust, and resistance. These responses further hinder data integration, perpetuating a self-reinforcing negative cycle.
- The Governance Stabilizer Loop (B1) shows how effective governance frameworks and ethical oversight enhance user trust in AI, encouraging adoption and improving system reliability. Greater confidence reduces resistance and the need for reactive governance changes, creating a stabilizing feedback mechanism.
- The Resistance Spiral Loop (R2) captures how fears around job displacement and skepticism toward automation intensify resistance to AI-KM initiatives. Reduced engagement limits the capture of tacit knowledge and adaptability, further compounding resistance in a downward spiral.
- The Training & Engagement Loop (B2) offers a counterbalancing dynamic, where investment in digital literacy and user training increases engagement, enhances trust, and improves the effectiveness of KM tools, especially those reliant on tacit knowledge inputs.
- The Capability-Scalability Loop (R3) highlights systemic disparities. Larger or more digitally mature organizations can dedicate greater resources to integration, training, and innovation, accelerating AI-KM benefits. However, this widens the capability gap between firms, reinforcing structural inequality.
- The Complexity Trap Loop (R4) underscores how increasing technological complexity, when poorly managed, leads to fragmented knowledge flows, reduced usability, and rising demand for new tools. Without strategic alignment, this leads to further complexity in a vicious cycle.

To clarify these interactions and identify leverage points, the CLD organizes variables into four subsystems:

- Technical Dynamics: Data quality, integration, and system complexity
- Human Dynamics: Resistance, training, and tacit knowledge engagement
- Organizational Structure: Size, resource availability, and digital maturity
- Governance & Strategic Alignment: Trust, ethics, and policy responsiveness

This systems-based perspective helps explain why isolated interventions, whether technical upgrades or training initiatives, often fail without coordinated attention to broader feedback dynamics. By identifying key loops and leverage points, the CLD provides a practical framework for designing more adaptive, equitable, and resilient AI-enabled KM strategies.

### 4.3 Leveraging AI and emerging technologies to address imposed challenges

This section of the chapter addresses the third research question by critically examining how organizations can leverage AI and emerging technologies to overcome the challenges identified in updating KM processes. The analysis highlights both technological solutions and socio-organizational dynamics and concludes with a practical implementation roadmap.

#### 4.3.1 Data and knowledge management strategies

Effectively managing large volumes of organizational knowledge depends on tools that go beyond manual or traditional information systems. Automated data processing such as categorization algorithms and filtering techniques plays a critical role in ensuring that relevant and timely information reaches decision-makers. Vladova et al. (2018) and Wielgórka (2023) emphasize that such automation supports more efficient identification of information gaps, helping organizations act on incomplete or overlooked knowledge. However, these systems primarily address explicit knowledge. Capturing tacit knowledge which is rooted in employee experience and often difficult to articulate remains a significant challenge. According to Schäffer et al. (2021), AI-based extraction tools and collaborative digital platforms can support the articulation of experiential knowledge by facilitating interaction, reflection, and annotation.

#### 4.3.2 Organizational and cultural interventions

Organizational culture can either support or hinder knowledge management (KM) transformation. Kianto et al. (2018) emphasize that trust and open communication are essential for reducing knowledge silos and encouraging collaboration across teams. While AI-based tools such as cross-functional collaboration platforms or network analysis algorithms can help surface hidden knowledge flows and support interaction across units, they cannot replace the need for planned, organization-specific efforts to shift cultural norms around sharing and learning. Practical challenges, including geographically dispersed teams, language barriers, and differences in local practices (Harrington et al., 2019; Gupta et al., 2022), require flexible approaches to knowledge adaptation and localization. Addressing these issues highlights the need to align technological solutions with human and contextual factors, rather than treating culture as an afterthought in KM initiatives.

#### 4.3.3 Leadership and workforce development

Leadership plays a critical role in integrating AI into knowledge management. Studies by Castellani et al. (2021) and Chang et al. (2017) highlight that transformational and ethical leadership help build a culture of knowledge sharing and reduce resistance to new technologies. The introduction of AI tools such as ChatGPT brings new challenges: excessive reliance on these tools may lead to skill loss among employees, while a lack of proper oversight can compromise the quality of knowledge produced (Retkowsky et al., 2024). Addressing these issues requires ongoing training and adjustments in job roles that combine human expertise with AI support. Therefore, leadership development initiatives should focus on fostering ethical decision-making and transparency to ensure responsible and effective use of AI in knowledge management.

#### 4.3.4 Technological solutions and AI integration

Building on the importance of leadership and workforce development, effective integration of AI technologies is essential to realize improvements in knowledge management. AI can streamline routine tasks, tailor knowledge delivery to users’ needs, and support real-time collaboration across teams. For example, Lei (2022) shows how cognitive computing can enhance knowledge transfer by identifying collaboration barriers, while Retkowsky et al. (2024) find that AI assistants improve information retrieval and help generate content efficiently. However, these benefits rely heavily on selecting appropriate tools, aligning AI capabilities with existing workflows, and ensuring systems can work together smoothly (Kawonga et al., 2023). To manage these complexities, organizations should implement AI solutions gradually and iteratively, allowing time to adjust processes and minimize operational disruption. Ultimately, the technical sophistication of AI tools must be balanced with flexible, adaptable processes to achieve meaningful gains in knowledge management.

Yet the effectiveness of such integration depends not only on internal readiness but also on the sectoral context shaping how AI and KM converge. In healthcare, for instance, Torres-Dela Cruz et al. (2019) describe how AI supports clinical decision-making by dynamically managing patient knowledge, though always under human supervision due to ethical and contextual considerations. By contrast, in energy-intensive manufacturing, Sanders et al. (2019) observe a more autonomous model, where AI-driven systems embedded with real-time sensing technologies optimize operational processes with minimal human input. These divergent patterns illustrate that AI–KM integration is not uniform: the degree of automation, the locus of decision-making, and the role of human expertise all shift according to domain-specific imperatives. Retkowsky et al. (2024) further complicate this picture by showing how generative AI tools are integrated bottom-up in office-based environments, reshaping individual workflows without formalizing KM processes at the organizational level. These variations highlight that realizing the benefits of AI in KM is not only a matter of tool selection or implementation strategy, but also of aligning technological possibilities with the epistemic and operational logic of the domain in which they are deployed.

#### 4.3.5 Ethical governance and sustainable KM

Ethical governance becomes essential as AI keeps re-shaping KM systems. Schäffer et al. (2021) and Leoni et al. (2022) highlight the importance of frameworks that promote transparency, fairness, and accountability to maintain trust among users and stakeholders. Protecting data privacy is a fundamental requirement. Algorithmic transparency allows stakeholders to audit AI decision-making, helping to uncover and address hidden biases (Safadi and Watson, 2023), while ongoing human oversight is necessary to ensure accountability in critical decision. Without these safeguards, organizations risk reputational damage and operational harm.

A forward-looking KM strategy integrates continuous learning and adaptability, recognizing that technological innovation alone cannot drive transformation (Retkowsky et al., 2024). The organizations to succeed must combine advanced data capabilities, organizational change management, leadership commitment, and ethical governance. They should balance automation with human expertise, appreciating that effective KM is as much about people and culture as it is about technology. This integrative approach positions organizations to build KM systems that are effective, accountable, and resilient during an ongoing technological and environmental shift.

**Figure 3:**
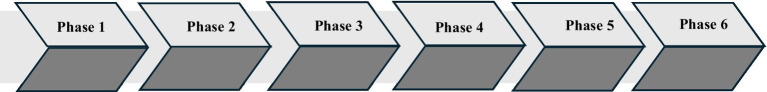
Phased implementation roadmap for AI-driven KM.

Building on the analysis above, the following roadmap (see Figure 3) translates these findings into a structured, phased implementation plan. Each phase addresses core challenges identified in knowledge strategy, infrastructure, culture, leadership, governance, and continuous improvement. The roadmap reflects both technical and human dimensions of AI-enhanced KM, offering a practical guide for organizations to navigate transformation with clarity and accountability.

##### 4.3.5.1 Objectives

**Table:** 

Phase 1	Phase 2	Phase 3	Phase 4	Phase 5	Phase 6
Assessment and strategic alignment	Infrastructure readiness and data preparation	Technology selection and pilot deployment	Cultural and workforce enablement	Governance, ethics, and scaling	Continuous improvement

##### 4.3.5.2 Key activities

**Table:** 

Phase 1	Phase 2	Phase 3	Phase 4	Phase 5	Phase 6
- Audit KM maturity. - Map critical knowledge processes. - Align KM with organizational strategy.	- Upgrade IT systems for interoperability. - Clean and organize existing data. - Add metadata and tags to improve searchability.	- Match AI tools to tasks (e.g., NLP, chatbots). - Run small pilots to assess fit. - Track usage, quality, and business value.	- Provide digital literacy training. - Set up KM communities or champions. - Facilitate open discussions about AI and job roles.	- Define ethical AI and data policies. - Use audits and feedback to monitor use. - Scale pilots thoughtfully across the org.	- Use AI to find gaps and outdated knowledge. - Adjust KM based on analytics and user input. - Foster a culture of ongoing learning.

##### 4.3.5.3 Expected outcomes

**Table:** 

Phase 1	Phase 2	Phase 3	Phase 4	Phase 5	Phase 6
Clear understanding of needs and priorities to guide focused KM efforts.	A reliable system with well-structured, accessible knowledge assets.	AI tools with demonstrated value and practical use cases.	Staff are engaged, informed, and ready to contribute to KM.	KM practices are consistent, ethical, and scalable.	KM remains relevant, efficient, and supports innovation.

This phased roadmap provides a practical structure for organizations to implement AI-enhanced KM in a manageable and adaptive way. Grounded in both technical feasibility and organizational readiness, it offers flexibility for sector-specific challenges while maintaining a consistent focus on strategic alignment, ethical governance, and continuous learning.

### 4.4 Theoretical contribution and propositions

This section introduces a conceptual framework based on the earlier analysis of how AI and emerging technologies influence organizational knowledge management. The framework integrates five core dimensions: technological drivers, KM processes, implementation challenges, strategic organizational responses, and anticipated outcomes. It positions AI as a transformative input that reshapes KM activities such as knowledge discovery, capture, sharing, and application, while emphasizing the sociotechnical factors that moderate this transformation.

**Figure 4:**
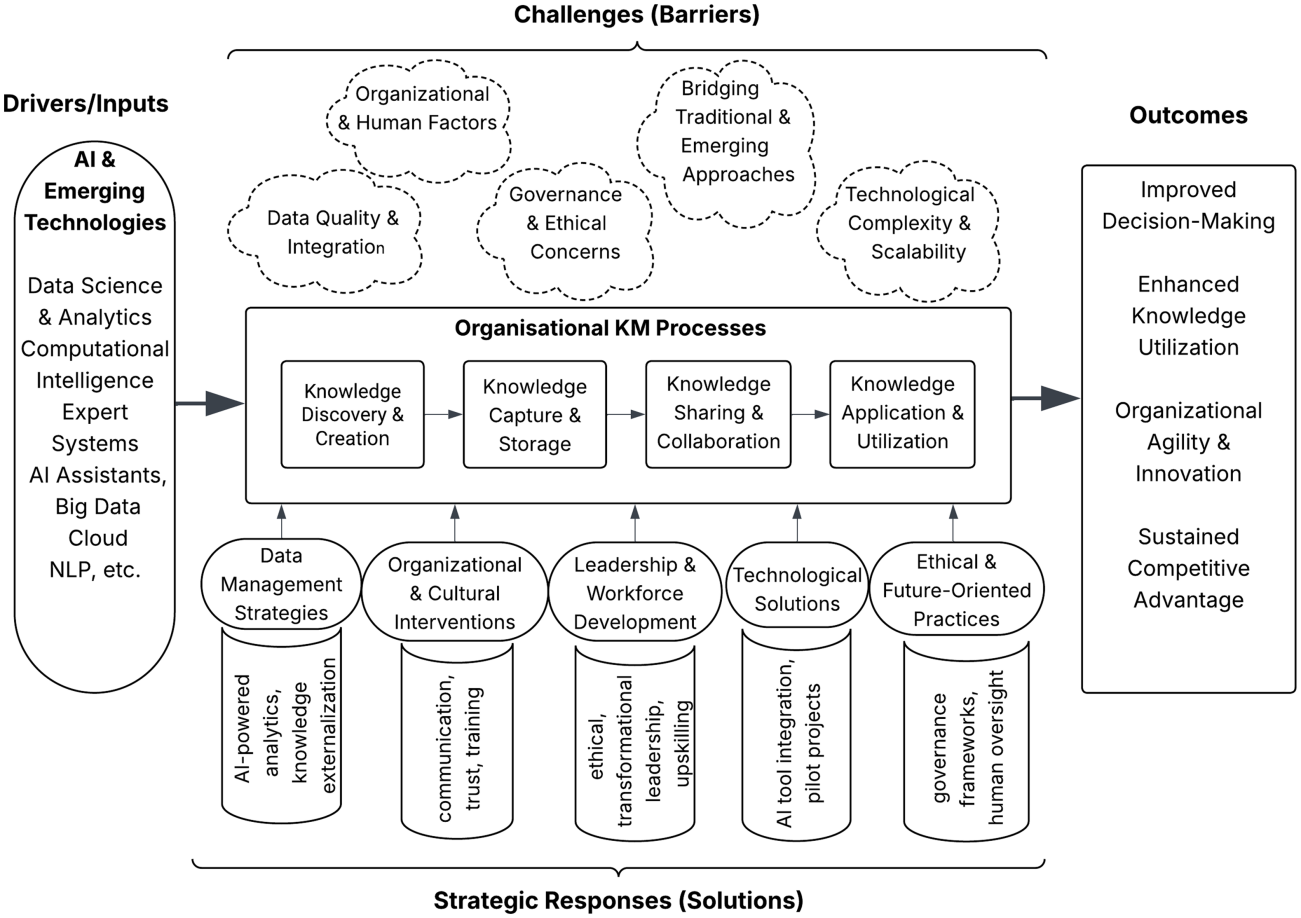
Conceptual framework for integrating AI and emerging technologies into organizational KM.

The following propositions operationalize this framework, translating its dimensions into empirically testable or practically actionable statements. Specifically, Propositions 1 and 2 address the technological drivers and data-related challenges depicted in the model. Propositions 3 and 4 correspond to the human and governance barriers, highlighting organizational culture and ethical oversight. Finally, Propositions 5 and 6 focus on strategic responses and structural adaptations that organizations can leverage to overcome these challenges, such as scalable AI solutions and hybrid knowledge management architectures. Figure 4 visually summarizes the framework, offering a reference point for researchers and practitioners seeking to design, implement, or evaluate AI-enabled KM strategies.

Proposition 1: Organizations with a culture open to innovation, strong real-time data capabilities, and mature digital infrastructure can use AI to turn static knowledge into a dynamic, evolving resource—making them more responsive and agile.

Proposition 2: When data is poor, systems are fragmented, and information comes in fast and varied forms, AI struggles to deliver useful insights. However, investing in data quality and system integration can significantly improve decision-making.

Proposition 3: If employees distrust AI, fear job loss, or lack digital skills (and the culture does not support change) AI-based KM systems are unlikely to succeed. Overcoming this requires targeted training and cultural support to encourage adoption.

Proposition 4: Transparent and inclusive AI governance within KM helps prevent bias, protect privacy, and avoid reputational harm. This builds trust and encourages long-term, responsible use of AI in organizations.

Proposition 5: When AI tools are scalable, easy to use, and fit specific industry needs, even smaller or less-resourced firms can benefit, if they also invest in digital skills, change readiness, and external support.

Proposition 6: Blending traditional KM methods, like communities of practice, with AI tools such as machine learning and NLP creates a hybrid system. This supports both structured and experience-based knowledge sharing—especially in a collaborative learning culture.

## 5 Conclusion and future work

The evolving relationship between organizational knowledge and technological innovation is reshaping the field of knowledge management. This systematic review has critically examined how AI and related technologies are influencing KM practices, drawing on evidence from 40 studies. The findings indicate that while AI enhances core KM activities including knowledge discovery, capture, sharing, and application, it also introduces new modes of collaboration, personalization, and decision support. However, the review also highlights that the integration of AI into KM is not straightforward. Significant challenges persist, including managing data quality and integration, overcoming organizational and human barriers, navigating governance and ethical complexities, and aligning emerging technologies with existing KM practices. These challenges can substantially limit the realization of AI’s potential benefits in organizational contexts. Addressing these barriers requires a multidimensional strategy: rigorous data management, attention to organizational culture and leadership, investment in workforce development, careful technology selection, and the implementation of robust ethical and governance frameworks. The conceptual framework proposed in this review (see Figure 4) offers an initial roadmap, though it requires further empirical validation.

Notably, the findings caution against viewing AI as a substitute for human expertise or established KM approaches. Instead, AI should be seen as a complement to human judgment, one that, when integrated thoughtfully, can strengthen organizational learning and adaptability. Ethical concerns, particularly around data privacy, algorithmic accountability, and human oversight, must remain central to both research and practice if AI-enabled KM is to advance in responsible and sustainable ways. This review is not without limitations. Its reliance on published literature may introduce selection bias and potentially overlooks emerging or practice-based innovations. Moreover, the diversity of organizational contexts and AI applications limits the generalizability of some findings.

To advance understanding in this area, future research should focus on several key topics identified in this review: First, as this study shows, knowledge management is evolving alongside how advanced systems access and process information in real time. Future research should examine knowledge flow models that balance real-time knowledge creation, validation, and application within operational settings. Second, the analysis highlighted the need for ethical governance frameworks that are flexible and continuously evolving to ensure responsible AI-KM practices. A key question to explore would be: how can organizations develop adaptive governance models that clearly define accountability and keep pace with rapidly changing technologies to provide ethical, transparent, and effective oversight? Third, there is a lack of research on the measurable costs and benefits of AI-enabled knowledge management, including investment needs and return on investment (ROI). Future studies should analyze these financial aspects by examining productivity, decision-making speed, and innovation outcomes.

Methodologically, these future studies should adopt mixed methods approaches that combine qualitative depth (e.g., case studies in diverse sectors, in-depth interviews) with quantitative validation (e.g., system analytics, performance metrics) and design science (model development and testing).

Together, these directions aim to deepen practical and theoretical understanding of AI-enabled knowledge management, guiding organizations toward more effective, responsible and sustainable implementations.

## Data Availability

The raw data supporting the conclusions of this article will be made available by the authors, without undue reservation.
