# Correction: Navigating the AI revolution: challenges and opportunities for integrating emerging technologies into knowledge management systems. Systematic literature review

**DOI:** 10.3389/frai.2025.1664837

**Published:** 2025-07-25

**Authors:** Teona Gelashvili-Luik, Peeter Vihma, Ingrid Pappel

**Affiliations:** ^1^Department of Software Science, Tallinn University of Technology, Tallinn, Estonia; ^2^Ragnar Nurkse Department of Innovation and Governance, Tallinn University of Technology, Tallinn, Estonia

**Keywords:** artificial intelligence, emerging technologies, knowledge management, organizational performance, digital transformation

Affiliation Department of Software Science, Tallinn University of Technology, Tallinn, Estonia was erroneously given to Peeter Vihma. The correct affiliation is Ragnar Nurkse Department of Innovation and Governance, Tallinn University of Technology, Tallinn, Estonia.

Affiliation Ragnar Nurkse Department of Innovation and Governance, Tallinn University of Technology, Tallinn, Estonia was erroneously given to Ingrid Pappel. The correct affiliation is Department of Software Science, Tallinn University of Technology, Tallinn, Estonia.

The original version of this article has been updated.

