# Supplementary material for: Navigating the AI revolution: challenges and opportunities for integrating emerging technologies into knowledge management systems. Systematic literature review
Source: Front Artif Intell. 2025 Jul 4;8:1595930. doi: 10.3389/frai.2025.1595930 (PMC12271129; doi:10.3389/frai.2025.1595930)
Supplement: Supplementary file 1 [file Data_Sheet_1.docx]

Supplementary Material

# Supplementary Tables and Figures

## Supplementary Tables

Supplementary Table 1. Inclusion and Exclusion Criteria

| Inclusion Criteria | Exclusion Criteria |
| --- | --- |
| Relevance: Studies focusing on the relationship between knowledge management (KM) and organizational performance. | **Relevance:** Studies not directly relevant to the intersection of KM, AI, and emerging technologies in organizational settings. |
| Impact on Knowledge Processes: Studies addressing the impact of digitalization, artificial intelligence (AI), and emerging technologies on KM practices. | **Focus:** Studies focusing solely on the public sector or government organizations. |
| Challenges and Strategies: Studies examining the challenges and strategies for updating existing KM processes to align with current trends. | **Specificity:** Studies do not address the specific research questions outlined in the Introduction section. |
| Variability Across Industries/Sizes: Studies exploring variations across industries and organizational sizes. | **Non-English Literature:** Non-English studies. |
| Language: Studies published in the English language. |  |

**Supplementary Table 2.** Quality Assessment Criteria

| Criterion | Description |
| --- | --- |
| 1. Clarity of Aims | Are the research objectives clearly stated and aligned with the study design? |
| 2. Methodology Appropriateness | Is the methodology (qual/quant/mixed) justified and suitable for the aims? |
| 3. Data Collection Transparency | Are data collection methods described in sufficient detail? |
| 4. Analysis Rigor | Is the analysis process transparent, systematic, and reproducible? |
| 5. Bias Consideration | Does the study address potential biases or limitations? |
| 6. Relevance to SLR Focus | How directly does the study address AI/emerging tech in KM? |
| 7. Technological Context | Does the study adequately describe the AI/emerging technology used (e.g., NLP, machine learning)? |

**Table 3.** Summary of Technology Categories and Their Contributions to Knowledge Management

| Technology/Category | Representative Studies (Year) | Study Type(s) | Main KM Process(es) | Key Insights |
| --- | --- | --- | --- | --- |
| Generative AI & AI | Retkowsky et al. (2024); Stollberg et al. (2004); Iaia et al. (2024); Stohr et al. (2024); Safadi & Watson (2023); Leoni et al. (2022); Schäffer et al. (2021); Jia et al. (2012) | Empirical, Conceptual, Technical | Knowledge transfer, sharing, creation, retention, collaboration, structuring | AI and generative models (e.g., ChatGPT) transform knowledge work, collaboration, and sharing; frameworks and empirical studies highlight risks, mechanisms, and implementation guidance. |
| Neural Networks & Hybrid AI | Liebowitz (2001); Kawonga et al. (2023); Sanders et al. (2019); Herrero et al. (2016); Miradi et al. (2009) | Empirical, Conceptual, Technical | Knowledge capture, discovery, diagnosis, integration, competitiveness | Neural networks and hybrid AI support knowledge discovery, scaling, and decision support across industries. |
| Fuzzy Logic & Machine Learning (ML) | Fu et al. (2022); Grzeszczyk (2021); Anshari et al. (2023); Vladova et al. (2018) | Empirical, Conceptual, Technical | KM system modeling, collecting, sharing, discovery | Fuzzy logic and ML enhance KM system modeling, knowledge collection, and automation |
| Big Data & Data Mining | Shaqrah & Alzighaibi (2023); Abualoush (2025); Sumbal et al. (2021); Thomas & Chopra (2020); Safhi et al. (2019); Khan & Vorley (2017); Depeige & Doyencourt (2015); Gullo (2015); Mahmood (2019); Natek & Zwilling (2014); Nguyen et al. (2014); Alonso et al. (2012) | Empirical, Conceptual, Technical | Acquisition, sharing, discovery, application, workflow optimization | Big data and data mining drive knowledge acquisition, discovery, and application, especially in healthcare, and education. |
| Other Digital Technologies | Wielgórka (2023); Kane (2017); Bandaru et al. (2017); Ristoski & Paulheim (2016); Bianchi et al. (2016); Braun et al. (2016); Santoro et al. (2018); Pisoni et al. (2024); Ashish et al. (2021) | Empirical, Conceptual | KM capacity, innovation, collaboration, knowledge integration | Internet of Thing (IoT), cloud, social media, and analytics tools foster innovation, collaboration, and digital knowledge integration in diverse sectors. |

**Supplementary Table 4.** Evolution of AI in Knowledge Management: Technological and Theoretical Shifts

| Period | Technological Paradigm | Knowledge Focus | Organizational Impact | Theoretical Orientation |
| --- | --- | --- | --- | --- |
| 1980s–1990s | Expert Systems (Rule-based KM) | Explicit, Codified Knowledge | Task Automation, Decision Support | Codified Knowledge, Stability, SECI |
| 2000s | Ontology and NLP (Semantic KM) | Metadata, Structured Texts | Structured Access, Retrieval Improvement | SECI, Early Dynamic Capabilities |
| 2010s | Machine Learning and Predictive Analytics (Adaptive KM) | Tacit and Contextual Knowledge | Adaptive Reasoning, Real-Time Feedback | Dynamic Capabilities, Organizational Learning |
| 2020s | Conversational AI and Generative Models (Embedded KM) | Cognitive Processes, Interaction, Tacit and Explicit Knowledge | Human–AI Collaboration, Embedded Knowledge Assistance | Dynamic Capabilities, Organizational Learning, Distributed Cognition |

**Appendix A. Publications Included in the Review**

**Supplementary Table A1.** Summary of Publications Included in the Systematic Literature Review

Abbreviations: Emp. = Empirical, Conc. = Conceptual, Tech. = Technical; KM Process: KC = Knowledge Creation, KS = Knowledge Sharing, KT = Knowledge Transfer, KD = Knowledge Discovery.The search string used was: (“knowledge management technology” OR “knowledge management tools” OR “knowledge management processes”) AND (“intelligent systems” OR “emerging technologies” OR “digitalization” OR “artificial intelligence”) AND “organization” AND (“adoption” OR “drivers” OR “strategies” OR “challenges” OR “success factors”) AND “innovation” NOT (“public sector” OR “government”)

| Tech/Category | Authors (Year) | Study Type | Context | KM Process |
| --- | --- | --- | --- | --- |
| Generative AI | Retkowsky et al. (2024) | Emp. | Knowledge work/Netherlands | KT, KS, KC, KR |
| AI | Stollberg et al. (2004) | Conc. | General/IT, Austria | Knowledge Collection |
| Neural Networks | Liebowitz (2001) | Conc. | General, N/A | KC, KS |
| AI in Business Communication | Iaia et al. (2024) | Emp. | Business, N/A | KS |
| AI in Predictive Maintenance | Stohr et al. (2024) | Emp | Manufacturing, Germany | Knowledge Building, Integration |
| AI | Safadi & Watson (2023) | Conc. | General, N/A | KS |
| AI in Manufacturing | Leoni et al. (2022) | Emp. | Manufacturing, Italy | Application, Utilization, Mediation |
| Knowledge-Based Systems in Engineering | Schäffer et al. (2021) | Conc. | Manufacturing/Automation, Germany/Denmark | Knowledge Structuring and Utilization, Knowledge Engineering |
| AI | Jia et al. (2012) | Conc. | Tourism, N/A | Crisis KM/KS or Tacit to Explicit Conversion |
| Neural Networks, Big Data | Kawonga et al. (2023) | Emp | General/IT, N/A | Scaling KM, Data Processing |
| Hybrid AI systems | Sanders et al. (2019) | Emp. | Energy-intensive manufacturing, N/A | KD |
| Hybrid AI | Herrero et al. (2016) | Emp. | Cross-sectoral (Electrical, Telecom, Food, Academies), N/A | Knowledge Analysis, KM Diagnosis, Decision Support, Competitiveness |
| Neural Networks | Miradi et al. (2009) | Tech. | Civil Engineering, Netherlands | KD |
| Fuzzy Logic/Theory | Fu et al. (2022) | Tech/Emp. | Enterprise level/General, N/A | KM System Modeling |
| Fuzzy Logic/Theory | Grzeszczyk (2021) | Emp. | IT, N/A | Knowledge Collecting/Sharing |
| Morphological tableau (a creativity/analysis tool) | Vladova et al. (2018) | Conc. | General Enterprise-wide, N/A | KS |
| Machine Learning | Anshari et al. (2023) | Conc. | General Business & Management, N/A | KD |
| Data Mining | Nohuddin & Zainol (2020) | Emp. | Peacekeeping/Defense, N/A | KD |
| Deep Learning | Obembe & Obembe (2020) | Conc. | General, N/A | Tacit KT |
| Big Data | Shaqrah & Alzighaibi (2023) | Emp. | Industrial Enterprises, Saudi Arabia | Acquisition, KS, Emission |
| Big Data | Abualoush (2025) | Emp. | Healthcare, Jordan | Acquisition, KS, and application |
| Big Data | Sumbal et al. (2021) | Emp. | Oil & Gas Industry, Pakistan | KC, KS, and Application |
| Big Data | Thomas & Chopra (2020) | Conc. | General, N/A | Broad KM themes including processing of tacit/explicit knowledge and decision-making |
| Big Data | Safhi et al. (2019) | Conc. | General, N/A | KD |
| Big Data | Khan & Vorley (2017) | Conc. | General, N/A | KD and decision support |
| Big Data | Depeige & Doyencourt (2015) | Conc. | Professional/Scientific/Technical Services, N/A | KM in cloud environments |
| Big Data | Gullo (2015) | Conc. | IT, N/A | KD |
| Data Mining | Mahmood (2019) | Emp. | Fgeneral Business sector, N/A | Knowledge Application & KD |
| Data Mining | Natek & Zwilling (2014) | Emp. | Education, N/A | KD |
| Big Data/Data Mining | Nguyen et al. (2014) | Tech. | General, N/A | KM Workflow Optimization |
| Data Mining | Alonso et al. (2012) | Emp. | Expert Systems, N/A | Knowledge Cooperation |
| ICT, Cloud Computing, Social Media | Wielgórka (2023) | Emp. | SME Enterprises, Poland | Knowledge acquisition, dissemination, use |
| Cloud Computing | Kane (2017) | Conc. | General/Cross-industry, N/A | KS, collaboration, KC, KT |
| Data mining | Bandaru et al. (2017) | Conc. | Cross industry, N/A | KD, KC, KT |
| Linked Open Data | Ristoski & Paulheim (2016) | Emp. | Cross industry domain, Germany | KD, knowledge integration, feature generation, data enrichment, interpretation |
| Call Data Records (CDR) analysis | Bianchi et al. (2016) | Emp. | Telecommunications, Ivory Coast | KD, pattern recognition, profiling, KC |
| Social Network Analysis | Braun et al. (2016) | Conc. | Social Networks / Data Science, N/A | KD |
| IoT | Santoro et al. (2018) | Emp. | Manufacturing/ Service sectors, Italy | KC, KS, and management capacity |
| Big Data, Data Analytics, AI | Pisoni et al. (2024) | Emp. | Financial Technology (FinTech), N/A | Knowledge acquisition, KS, application |
| AI | Ashish et al. (2021) | Emp. | Information Technology (IT), Global | KS, talent management, innovation strategy |

**Appendix B. Study Quality Assessment Criteria**

**Supplementary Table B1.** Quality Assessment of Included Studies

| Study (Author, Year) | 1. Aims | 2. Methodology | 3. Data Collection | 4. Analysis | 5. Bias | 6. Relevance | 7. Tech Context | Total Score | Quality Rating |
| --- | --- | --- | --- | --- | --- | --- | --- | --- | --- |
| Liebowitz (2001) | 2 | 1 | 1 | 1 | 1 | 2 | 1 | 9 | Medium |
| Stollberg et al. (2004) | 1 | 1 | 0 | 1 | 1 | 2 | 2 | 8 | Medium |
| Miradi et al. (2009) | 2 | 2 | 2 | 2 | 2 | 2 | 2 | 14 | High |
| Jia et al. (2012) | 2 | 2 | 0 | 0 | 0 | 1 | 2 | 7 | Low |
| Alonso et al. (2012) | 2 | 2 | 2 | 2 | 0 | 2 | 2 | 12 | High |
| Natek & Zwilling (2014) | 2 | 2 | 1 | 2 | 2 | 2 | 1 | 12 | High |
| Nguyen et al. (2014) | 2 | 2 | 2 | 2 | 2 | 2 | 2 | 14 | High |
| Depeige & Doyencourt (2015) | 2 | 2 | 1 | 1 | 2 | 2 | 2 | 12 | High |
| Gullo (2015) | 1 | 1 | 1 | 0 | 0 | 2 | 2 | 7 | Low |
| Herrero et al. (2016) | 2 | 2 | 2 | 2 | 1 | 2 | 2 | 13 | High |
| Ristoski & Paulheim (2016) | 2 | 2 | 2 | 2 | 1 | 1 | 2 | 12 | High |
| Bianchi et al. (2016) | 2 | 2 | 1 | 1 | 2 | 2 | 2 | 12 | High |
| Braun et al. (2016) | 2 | 2 | 2 | 2 | 1 | 2 | 2 | 13 | High |
| Khan & Vorley (2017) | 2 | 2 | 2 | 1 | 1 | 2 | 2 | 12 | High |
| Kane (2017) | 2 | 2 | 1 | 1 | 0 | 1 | 1 | 8 | Medium |
| Bandaru et al. (2017) | 2 | 2 | 2 | 1 | 1 | 2 | 2 | 12 | High |
| Vladova et al. (2018) | 1 | 2 | 1 | 1 | 1 | 1 | 1 | 8 | Medium |
| Santoro et al. (2018) | 2 | 2 | 2 | 2 | 2 | 2 | 2 | 14 | High |
| Sanders et al. (2019) | 2 | 2 | 2 | 1 | 1 | 2 | 2 | 12 | High |
| Mahmood (2019) | 2 | 2 | 1 | 1 | 2 | 2 | 2 | 12 | High |
| Safhi et al. (2019) | 2 | 2 | 1 | 2 | 1 | 2 | 2 | 12 | High |
| Thomas & Chopra (2020) | 2 | 2 | 2 | 1 | 1 | 2 | 2 | 12 | High |
| Nohuddin & Zainol (2020) | 2 | 2 | 2 | 2 | 1 | 1 | 2 | 12 | High |
| Obembe & Obembe (2020) | 2 | 2 | 1 | 1 | 2 | 2 | 2 | 12 | High |
| Schäffer et al. (2021) | 2 | 2 | 1 | 2 | 1 | 2 | 2 | 12 | High |
| Grzeszczyk (2021) | 2 | 2 | 2 | 1 | 1 | 2 | 2 | 12 | High |
| Sumbal et al. (2021) | 2 | 2 | 2 | 2 | 0 | 2 | 2 | 12 | High |
| Leoni et al. (2022) | 2 | 2 | 1 | 2 | 2 | 1 | 2 | 12 | High |
| Safadi & Watson (2023) | 2 | 1 | 1 | 1 | 1 | 1 | 1 | 8 | Medium |
| Kawonga et al. (2023) | 2 | 2 | 2 | 2 | 1 | 2 | 2 | 13 | High |
| Anshari et al. (2023) | 1 | 1 | 1 | 1 | 1 | 2 | 1 | 8 | Medium |
| Shaqrah & Alzighaibi (2023) | 2 | 1 | 1 | 2 | 2 | 2 | 2 | 12 | High |
| Wielgórka (2023) | 2 | 2 | 2 | 1 | 1 | 2 | 1 | 12 | High |
| Leoni et al. (2024) | 2 | 2 | 2 | 2 | 2 | 2 | 2 | 14 | High |
| Stohr et al. (2024) | 2 | 2 | 2 | 2 | 2 | 1 | 2 | 13 | High |
| Iaia et al. (2024) | 2 | 2 | 2 | 2 | 1 | 1 | 2 | 12 | High |
| Retkowsky et al. (2024) | 2 | 2 | 2 | 2 | 1 | 2 | 1 | 12 | High |
| Pisoni et al. (2024) | 2 | 2 | 2 | 2 | 2 | 2 | 2 | 14 | High |
| Yadegari and Mohammadi (2024) | 2 | 2 | 2 | 2 | 1 | 2 | 2 | 13 | High |
| Abualoush (2025) | 2 | 2 | 2 | 2 | 1 | 2 | 2 | 13 | High |

## Supplementary Figures

**Identification**

Records identified through database searches (n=1568)

Total records (n=1568)

Duplicates removed (n=13)

Additional records identified through other searches and snowballing (n=12)

**Screening**

Records screened (n=1555)

Records excluded (n=1218)

**Eligibility**

Full-text articles assessed for eligibility (n=337)

**Studies included in SLR (n=40)**

Full-text articles excluded with reasons (n=297)

**Supplementary Figure 1.** PRISMA flow diagram illustrating the study selection process.


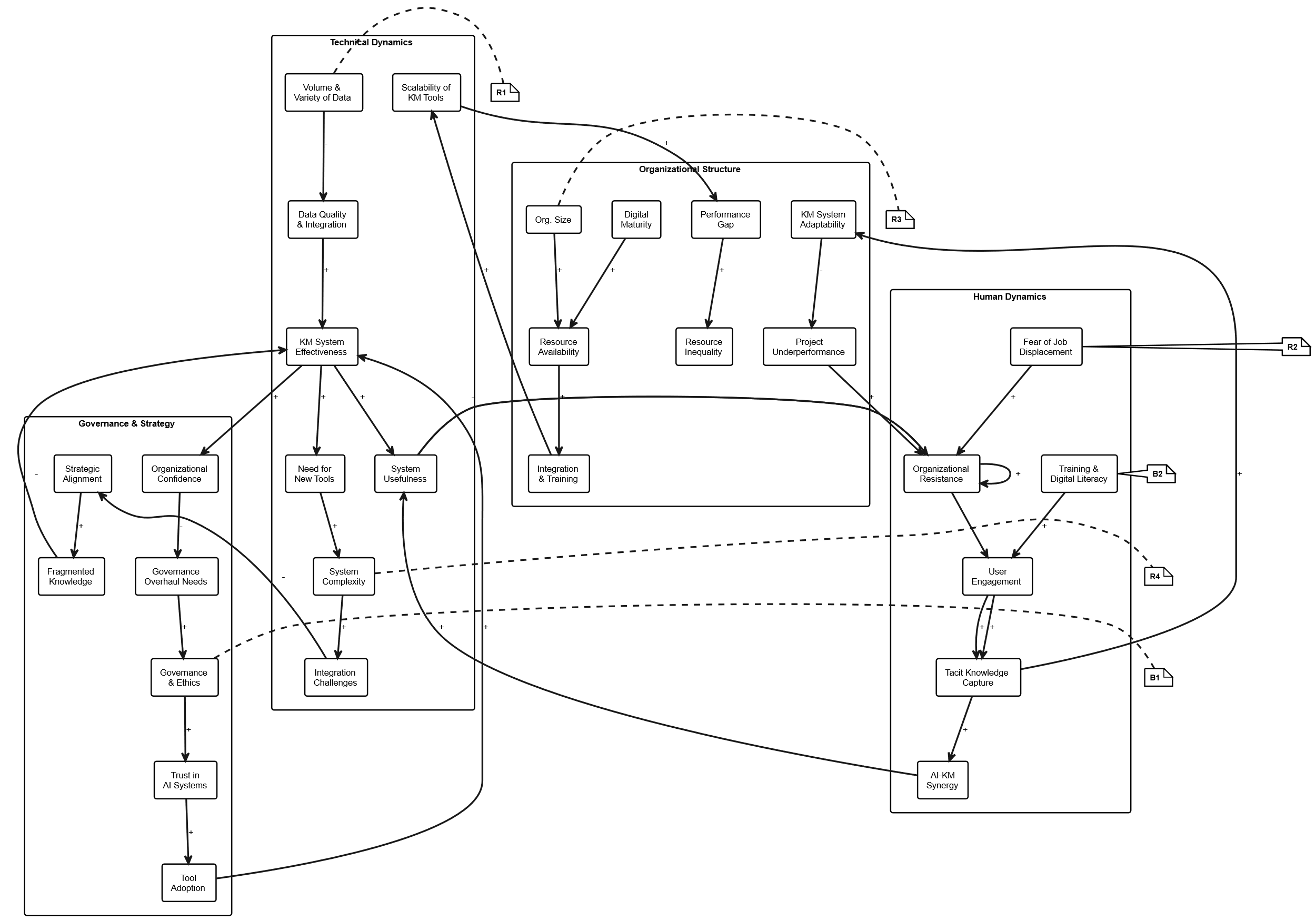


Supplementary Figure 2. Causal Loop Diagram.

**Phase 6**

**Phase 5**

**Phase 4**

**Phase 1**

**Phase 3**

**Phase 2**

Gunae big

Gunae big

Gunae big

**Objectives**

| Phase 1 | Phase 2 | Phase 3 | Phase 4 | Phase 5 | Phase 6 |
| --- | --- | --- | --- | --- | --- |
| *Assessment and Strategic Alignment* | *Infrastructure Readiness and Data Preparation* | *Technology Selection and Pilot Deployment* | *Cultural and Workforce Enablement* | *Governance, Ethics, and Scaling* | *Continuous Improvement* |

**Key Activities**

| Phase 1 | Phase 2 | Phase 3 | Phase 4 | Phase 5 | Phase 6 |
| --- | --- | --- | --- | --- | --- |
| - Audit KM maturity.  - Map critical knowledge processes.  - Align KM with organizational strategy. | - Upgrade IT systems for interoperability.  - Clean and organize existing data.  - Add metadata and tags to improve searchability. | - Match AI tools to tasks (e.g., NLP, chatbots).  - Run small pilots to assess fit.  - Track usage, quality, and business value. | - Provide digital literacy training.  - Set up KM communities or champions.  - Facilitate open discussions about AI and job roles. | - Define ethical AI and data policies.  - Use audits and feedback to monitor use.  - Scale pilots thoughtfully across the org. | - Use AI to find gaps and outdated knowledge.  - Adjust KM based on analytics and user input.  - Foster a culture of ongoing learning. |

**Expected Outcomes**

| Phase 1 | Phase 2 | Phase 3 | Phase 4 | Phase 5 | Phase 6 |
| --- | --- | --- | --- | --- | --- |
| Clear understanding of needs and priorities to guide focused KM efforts. | A reliable system with well-structured, accessible knowledge assets. | AI tools with demonstrated value and practical use cases. | Staff are engaged, informed, and ready to contribute to KM. | KM practices are consistent, ethical, and scalable. | KM remains relevant, efficient, and supports innovation. |

Supplementary Figure 3. Phased Implementation Roadmap for AI-driven KM


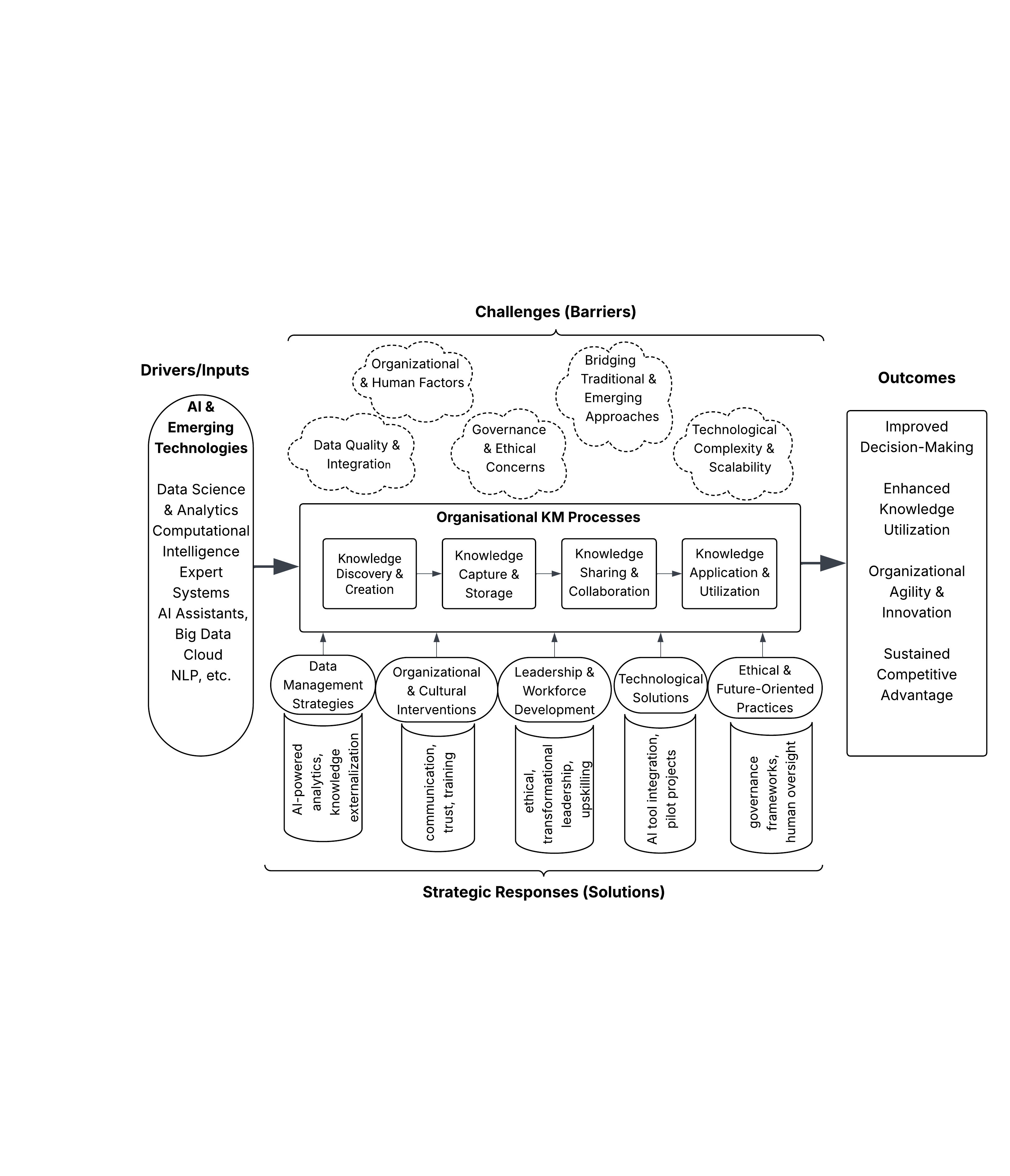


**Supplementary Figure 4.** Conceptual Framework for Integrating AI and Emerging Technologies into Organizational Knowledge Management
